# An improved ovitrap-based surveillance framework: facilitating cost-efficient monitoring and efficacy assessment of integrated vector management strategies for dengue outbreak control

**DOI:** 10.1186/s13071-025-07002-8

**Published:** 2025-09-24

**Authors:** Xiang Guo, Shihan Liu, Xiaohua Liu, Kaihao Chen, Wushen Chen, Zhenyu Huang, Ziyao Li, Shu Zeng, Haiyang Chen, Qing He, Liu Ge, Yijia Guo, Xiaming Chen, Zhiqiang Peng, Benyun Shi, Jiming Liu, Xiao-Guang Chen, Xiaohong Zhou

**Affiliations:** 1 Institute of Tropical Medicine, Department of Pathogen Biology, School of Public Health, Southern Medical University; Guangdong Provincial Key Laboratory of Tropical Disease Research; Key Laboratory of Prevention and Control for Emerging Infectious Diseases of Guangdong Higher Institutes; Key Laboratory of Infectious Diseases Research in South China, Ministry of Education, Guangzhou, 510515 Guangdong China; 2 School of Basic Medical Sciences, Henan University, Kaifeng, 475004 China; 3 Luohu Center for Disease Control and Prevention, Shenzhen, 518012 China; 4 Bao’an Center for Disease Control and Prevention, Shenzhen, 518101 China; 5 Public Health Service Center, Bao’an District, Shenzhen, 518126 China; 6 Guangdong Provincial Center for Disease Control and Prevention, Guangzhou, 511430 China; 7 College of Computer and Information Engineering, Nanjing Tech University, Nanjing, 211816 China; 8 Department of Computer Science, Hong Kong Baptist University, Hong Kong, 999077 China

**Keywords:** *Aedes albopictus*, Surveillance system, Oviposition trap, Dengue outbreak, Integrated mosquito vector management

## Abstract

**Background:**

Dengue fever, transmitted primarily by Aedes aegypti and Ae. albopictus, remains one of the most pervasive mosquito-borne diseases worldwide. In China, the mosquito oviposition trap (MOT) - based Aedes monitoring system has become a cornerstone for dengue prevention and control. However, during outbreaks, this system faces operational challenges because of its labour-intensive nature and time requirements, limiting its efficiency for rapid vector control assessment.

**Methods:**

Based on the oviposition behavior of Ae. albopictus, a novel Improved Ovitrap (IMT) was designed, featuring a bucket-shaped body and a thermoplastic elastomer (TPE) oviposition band. Two field investigations were conducted in Guangzhou, Guangdong Province, China. Field Investigation 1 focused on continuous mosquito surveillance to evaluate the effectiveness of the IMT. Distance-incremental spatial autocorrelation analysis was performed to determine the monitoring radius of the IMTs, and suitable sampling fractions were estimated to identify the optimal sampling density. Field Investigation 2 aimed to compare the monitoring effectiveness of the IMT and the standard MOT for Ae. albopictus. Finally, an IMT-based surveillance strategy was proposed for local dengue epidemic control and was preliminarily implemented within case-area targeted intervention (CATI) practices.

**Results:**

Our research established a significant positive correlation between the newly developed new Ovitrap Index (NOI) and the existing mosquito or oviposition positive index (MOI), which facilitated the creation of a IMT based surveillance strategy for dengue outbreak response. This optimized system recommends deploying six IMTs per standard 120,000 m2 CATI zone, maintaining continuous 24-hour monitoring cycles until official outbreak resolution, and implementing NOI threshold categories (0, 0-10, 10-20, 20-40, and ≥40) analogous to established MOI standards. Following successful implementation during three 2024 Guangdong CATI initiatives, this strategy has proven adaptable to complex urban environments while providing daily surveillance capabilities superior to those of conventional MOI-based systems.

**Conclusions:**

In the present study, the IMT was developed and evaluated for field surveillance of Ae. albopictus mosquitoes. The core usage parameters of the IMT-based surveillance system, including the working radius and area deployment density, have been determined using systematic field investigations combined with mathematical modelling assessments. Furthermore, a novel strategy utilizing the IMT for evaluating the efficacy of integrated mosquito vector management in CATI during dengue outbreaks has been proposed. Preliminary results have confirmed the feasibility of using the IMT at outbreak sites, providing valuable support for CATI-based implementation. This innovative monitoring system offers an alternative solution and implementation strategy for conducting cost-effective surveillance of the dengue vector Ae. albopictus, as well as for evaluating the efficacy of integrated vector management during outbreaks.

**Graphical Abstract:**

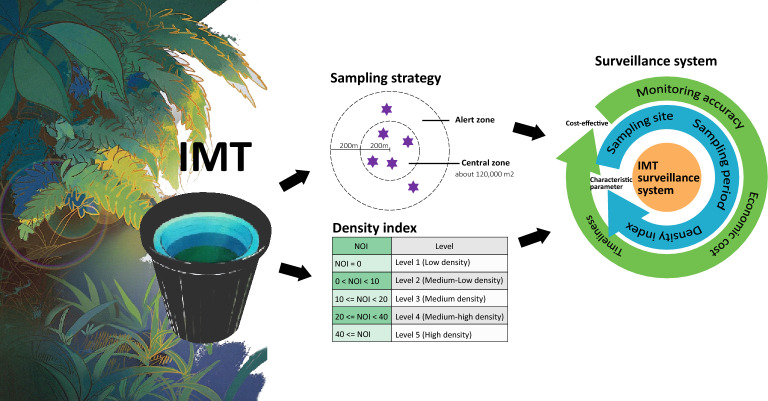

**Supplementary Information:**

The online version contains supplementary material available at 10.1186/s13071-025-07002-8.

## Background

Dengue, caused by the dengue virus (DENV), is one of the most widespread mosquito-borne diseases, especially in tropical and subtropical regions, with over half of the world’s population residing in dengue-risk areas [1, 2]. The global burden of dengue has increased dramatically in recent decades owing to complex factors including global warming and the expanded habitats of dengue vectors, notably *Aedes aegypti* and *Ae. albopictus* [3–6]. By August 2024, the number of reported dengue cases worldwide surpassed 12.3 million [7]. Since the re-emergence of a dengue outbreak in Foshan, Guangdong Province, in 1978, dengue has been remained prevalent in southern China, particularly in Guangdong, Yunnan, Fujian, and Zhejiang Provinces [8].

Despite some advancements in the development of novel vaccines and enhanced case management [9–11], current dengue control strategies heavily focus on the surveillance and control of *Aedes* mosquito vectors. Oviposition traps (OTs), first developed by Fay and Eliason in 1966 [12], are artificial containers, typically small, dark-colored plastic containers filled with water and an attractant-infused substrate where female mosquitoes deposit their eggs [13]. The World Health Organization (WHO) recommended the use of ovitraps for monitoring *Ae. albopictus* or *Ae. aegypti* mosquitoes in 1994 [14]. Compared with other surveillance techniques, such as human landing collections (HLCs) or human-baited double net traps (HDNs), ovitraps offer several advantages, including cost-effectiveness, ease of deployment, and noninvasiveness [15–17]. In general, a limited number of ovitraps are adequate for determining the presence of vectors. Furthermore, fewer than 100 ovitraps can reliably assess the population density in a large urban community [18]. Moreover, it is convenient to analyze data collected from ovitraps just by calculating the percentage of positive traps with deposited eggs and the mean number of eggs per trap [15–17].

On the basis of the features and advantages of OTs, a series of improved tools, including gravid ovitraps and BG-Gravid Aedes Traps (BG-GATs), have been developed to refine trap designs, augment breeding habitats, and improve mosquito capture efficiency [19–21]. Specifically, the mosquito oviposition trap (MOT), an improved tool derived from the OTs, has been utilized in field surveillance in China since 2005 to measure the mosquito or oviposition positive index (MOI) (developed from the ovitrap positivity index, OPI) of wild *Ae. albopictus* populations [22]. This unique device is strategically positioned in well-lit shaded areas or at a maximum height of 1 m from the ground, focusing on green belts, grassy areas, and shaded spots where mosquitoes and other insects are known to inhabit. The recommended deployment involves positioning one trap every 50–100 m for a continuous 4-day period [23]. On the fourth day, field researchers should carefully examine and collect both adult mosquitoes and mosquito eggs from the trap, documenting the number of positive traps for *Aedes* mosquitoes (adults or eggs), as well as the total count of captured adult mosquitoes [23]. The MOI is calculated using the following formula: MOI = *N*_*u*_/*N*_*e*_ × 100, where *N*_*u*_ denotes the count of *Aedes* mosquitoes or (and) positive *Aedes* mosquito eggs in the deployed and collected MOTs for recycling, and N_e_ represents the number of effective MOTs collected post-deployment [23]. Duan et al. developed an epidemic forecast and phased response system for dengue control and prevention by comparing the relationship between the MOI and the Breteau index (BI), creating four response levels: level 1, MOI < 5; level 2, MOI 5–10; level 3, MOI 10–20; and level 4, MOI > 20 [24]. This MOT-based surveillance system has become an important methodology for *Aedes* surveillance programs across China.

Recently, field evaluations of the MOI system have demonstrated both its strengths and weaknesses. Although effective for surveillance, the current MOI protocol faces significant constraints in terms of substantial labor requirements and its demanding 4-day monitoring period. These challenges limit its field applicability, impeding its effective utilization, promotion, and assessment of disinfection and control efficacy in real-world scenarios. Issues concerning enhancements of MOT and its system have been consistently raised. Persistent calls for system optimization highlight two critical needs: (1) a reduction in trap deployment density and (2) a shortened monitoring duration without compromising the surveillance effect. To address these challenges, we developed an improved mosquito ovitrap (IMT) system to increase the sensitivity of *Ae. albopictus* surveillance, establish optimal deployment parameters (radius and density) across urban and rural settings through comparative field experiments with the MOT, and validate its real-world efficacy in dengue outbreak response as part of integrated vector management (IVM). Our results demonstrate that the IMT system provides a cost-effective, scalable solution for dengue prevention, with practical applicability in both surveillance and IVM efficacy assessment during epidemics.

## Methods

### IMT design specifications

The IMT design is fundamentally grounded in the oviposition behavior of *Ae. albopictus* and its adaptability to varying weather conditions and the subsequent management of collected eggs. Compared with conventional ovitrap models, this IMT design is characterized by a larger container volume (5 L; upper diameter: 215 mm, lower diameter: 165 mm, height: 245 mm; Fig. S1). This large volume of container was specifically made in black to create a more alluring breeding environment for *Ae. albopictus* [25] because of the preference of this species for dark-colored oviposition sites [26].

The oviposition carrier was deliberately designed to account for the oviposition behavior of *Ae. albopictus*, because these mosquitoes lay eggs on the container edge rather than directly in water, unlike *Ae. aegypti* [27]. To address the limitations of disposable filter papers in MOTs (e.g., powdery residue, cumbersome egg collection), the IMT incorporates a reusable, deep blue thermoplastic elastomer (TPE) oviposition strip (550 mm × 40 mm × 10 mm; Fig. S1). This oviposition strip features distinct surface characteristics: a smooth outer face for optimal bucket adhesion and an inner face with transverse stripes spaced 6.5 mm apart. When deployed, the strip forms a circular configuration with (1) the smooth exterior pressed against the container wall and (2) the textured interior floating at the water interface. This design provides an inclined oviposition surface that significantly reduces egg displacement into the water column.

The oviposition band/strip maintains optimal positioning relative to water level fluctuations in tropical/subtropical habitats across variable weather conditions. For deployment, (1) the container was filled with clean water to 40–50% capacity, and (2) the substrate was secured vertically along the inner wall to ensure continuous flotation on the water surface.

### Field study to evaluate the effectiveness of the IMT

#### Investigation areas

**Fig. 1:**
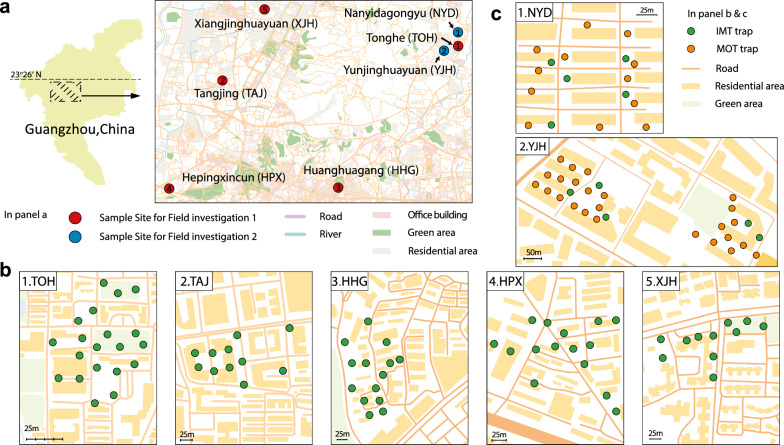
Investigation sites and the environments for evaluating the effectiveness of IMT in Guangzhou. **a** Two field investigations encompassing seven sites involving *Ae. albopictus* were conducted in Guangzhou. **b** Five sites and surrounding environments of field investigation 1. **c** Two sites and surrounding environments of field investigation 2

Two field investigations focusing on *Ae. albopictus* were conducted in Guangzhou, Guangdong Province, China (Fig. 1a). This city features a subtropical monsoon climate typically with four distinct seasons. Summers are characterized by high temperatures and heavy rainfall, whereas winters are mild and humid. With an annual mean temperature of 21.6 °C and an average annual rainfall of approximately 1980 mm, these conditions provide an ideal environment for the breeding and growth of *Ae. albopictus* mosquitoes.

For field investigation 1, we selected five sites, including Tonghe (TOH; 23.185734N, 113.334702E), Huanghuagang (HHG; 23.141904N, 113.298651E), Hepingxincun (HPX; 23.138515N, 113.237929E), Tangjing (TAJ; 23.167231N, 113.255696E), and Xiangjinghuayuan (XJH; 23.203722N, 113.267219E), were selected for field investigation 1 (Fig. 1a, b). HHG, HPX, TAJ, and XJH were residential areas, whereas TOH was a school area (Fig. 1b). For field investigation 2, two sites, Nanyidagongyu (NYD; 23.190295N, 113.335764E) and Yunjinghuayuan (YJH; 23.184511N, 113.329757E), were selected (Fig. 1a, c). Both NYD and YJH were residential areas (Fig. 1c).

#### Field investigation 1: continuous field mosquito surveillance to assess the effectiveness of IMT

A network of 71 IMTs was established across five investigation sites from July 2020 to October 2020 (Fig. 1b). The traps were placed in the mixed locations weekly. After 7 days, the samples were sent to our laboratory for assessment of the presence of mosquito eggs, larvae, and adults. To observe the distribution of mosquito eggs on the strip, manual counting was conducted using counters. A Pasteur pipette was used to rinse the eggs off the strip with clean water onto filter paper. Once the water had completely drained, the eggs were collected and stored at −20 °C for more than 24 h to ensure complete inactivation.

#### Field investigation 2: comparative monitoring effectiveness of IMT versus MOT for *Ae. albopictus*

We employed a spatially interspersed deployment strategy for the IMT and MOT traps within their natural habitats (Fig. 1c). Eggs were collected from September 2024 to December 2024 and subsequently transported to the laboratory for counting (Fig. 1c). The IMT and MOT traps were consistently placed in fixed locations each time. After 4 days of deployment, the traps were retrieved and sent to the laboratory for assessment of mosquito developmental stages.

### Distance incremental spatial autocorrelation analysis

Moran’s index (Moran’s I) quantifies the spatial similarity between neighboring units in space by assessing the correlation between their spatial locations and sample values. Specifically, it is computed as the product of the difference between each unit’s value and that of its adjacent units. As Moran’s I approaches the boundary value of its range, it signifies a stronger aggregation effect for the variable. Conversely, when Moran’s I deviates from this boundary value, it indicates a weaker aggregation effect.

To assess the presence of spatial autocorrelation (SA) in the IMT, we computed Moran’s I [28]. Moran’s I is a correlation coefficient that characterizes whether attribute values exhibit significant dispersion, randomness, or clustering in space [29]. The range of Moran’s I varies from −1 to 1, where negative values indicate a dispersed spatial pattern, 0 signifies a random pattern, and positive values denote a clustered pattern. The magnitude of Moran’s I reflects the strength of the spatial pattern, with larger positive values (closer to 1) suggesting more pronounced clustering of similar attribute values [29]. The Moran’s I value of 0.2 is a threshold for determining whether there is a correlation [29]. The formula for calculating Moran’s I is as follows:

$$ {\text{Moran}}^{\prime}{\text{s}}\frac{n}{S}\frac{{\sum\nolimits_{i = 1}^{n} {\sum\nolimits_{j = 1}^{n} {W_{i,j} Z_{j} Z_{j} } } }}{{\sum\nolimits_{j = 1}^{n} {z_{i}^{2} } }} $$,

where *n* is the number of features, *S*_0_ is the sum of all spatial weights, *i* and *j* denote two features, *w*_*i,j*_ is the spatial weight between those features, and *z* is the deviation of the attribute at that feature from the mean.

We performed incremental spatial autocorrelation analysis by calculating Moran’s I for IMTs across successive distance thresholds, beginning with 50-m intervals and systematically expanding the range by 10-m increments (50 m, 60 m, 70 m, etc.) until maximum sampling distance was covered.

The statistical significance of Moran’s I was determined by *P*-values or *z*-scores. By considering the number of pairwise IMTs within each bin and the variance between the expected and observed Moran’s I values, this tool calculates a *P*-value to determine if there is a statistically significant difference. The resulting Moran’s I and *P*-values at each incrementally larger distance bin enable us to identify the scale at which spatial autocorrelation occurs. The Moran’s *z*-score was calculated as the standardized value of the local Moran’s I index, which is used to assess whether the observed spatial pattern exhibits clustering, dispersion, or randomness.

### Estimation of the suitable sampling fractions

Although advanced point-selection algorithms exist (e.g., sequential selection, simulated annealing, and generalized random tessellation stratification), their complexity often limits their practical implementation. We therefore adopted a simplified sampling strategy, with layout optimization guided by relative error (RE) minimization in population extrapolation.

### IMT-based surveillance strategy for case-area targeted interventions (CATIs)

In resource-limited settings, the implementation of CATIs offers a strategic approach to outbreak control by concentrating efforts on areas with recent disease cases [30]. Technical guidance documents such as the “Technical Guidelines for Dengue Fever Prevention and Control” released by the Chinese Center for Disease Control and Prevention [31], and the “Professional Technical Guidelines for Dengue Fever Prevention and Control in Guangdong Province” issued by the Guangdong Provincial Health Commission [32], organize interventions across three concentric zones: core, alert, and surveillance. The core zone encompasses a 200-m radius around infected individuals’ residences or workplaces, corresponding to the typical flight range of *Aedes* mosquitoes. This central area is surrounded by an alert zone extending an additional 200 m outwards, with rural implementations covering natural villages progressing to administrative units as needed, whereas urban deployments typically include adjacent streets and neighborhoods. Beyond these areas, a surveillance zone is established with monitoring intensity adjusted according to seasonal transmission risks.

During CATI execution, broad investigations are conducted using a standardized questionnaire to document patient activities and mosquito-bite history. Research results are complemented by laboratory tests of patient serum and captured mosquitoes. Active case detection involves systematic searches with a 200-m core radius, including checks at local healthcare facilities. When warranted, serological surveys are performed to assess transmission dynamics, with preliminary epidemiological reports required within 24 h of case identification and follow-up updates as the investigation progresses. Intervention effectiveness is monitored using key indicators, including case incidence and mosquito density metrics, with outbreak resolution declared only after 21 consecutive days without new cases and confirmation of a Breteau index (BI) below 5.

In our study, we collaborated with the Shenzhen Luohu CDC to integrate IMTs into the CATI framework across the core and alert zones of two active transmission areas in Shenzhen, China. We collected mosquito eggs on a daily basis and identified their species, with continuous monitoring maintained until local health authorities officially concluded the intervention period.

## Results

### Dynamics of the wild population of *Ae. albopictus* monitored using the IMT surveillance network

**Fig. 2:**
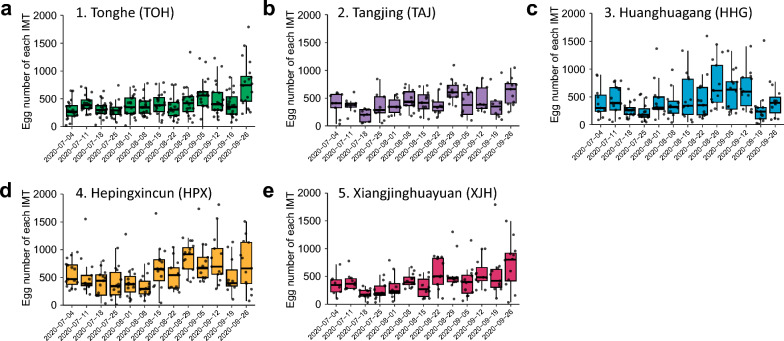
Seasonal dynamics of the wild population of *Ae*. *albopictus* monitored using IMTs. Five sites including Tonghe (**a**), Tangjing (**b**), Huanghuagang (**c**), Hepingxincun (**d**), and Xiangjinghuayuan (**e**) were involved

From July 2020 to October 2020 (spanning 13 weeks), a total of 416,800 eggs of *Ae. albopictus* were collected through the IMT surveillance network, with an average of 67.51 ± 51.91 (5–5500) eggs per IMT per week recorded (Fig. 2; Fig. S2). A significant increase in egg numbers was observed at the beginning of August, and the trend continued to increase, remaining elevated despite a few fluctuations, until peaking at 5500 eggs collected from one single IMT in HPX during the week of 20–26 September 2022 (Fig. 2; Fig. S2).

### The monitoring radius of IMTs determined using incremental spatial autocorrelation analysis

**Fig. 3:**
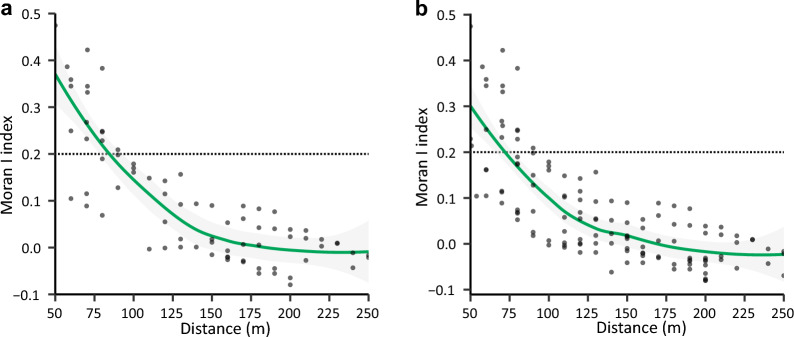
Dynamics of Moran's I values resulting from incremental spatial autocorrelation analysis, based on p-value thresholds of 0.1 (**a**) and 0.05 (**b**), respectively

In the analysis, two sets of values were selected from the results exhibiting spatial autocorrelation with *P* values of less than 0.1 and 0.05 (Fig. 3). The Moran’s *z*-score and *P*-value suggested that the spatial pattern of the observed data was influenced by spatial autocorrelation (Fig. S3). Interestingly, Moran’s I exhibited a typical decreasing trend, with a sharp initial decline that gradually stabilized above 150 m (Fig. 3). The point where the declining curve intersected a Moran’s I value of 0.2 occurred at approximately 75 m (Fig. 3). These results indicate a distinct spatial distribution pattern of mosquito eggs. Specifically, spatial autocorrelation was observed within 0–75 m, whereas beyond 150 m, the distribution overlapped with that of other ovipositing mosquitoes.

### Revealing the sampling density by evaluating variations in the relative across sampling fractions

**Fig. 4:**
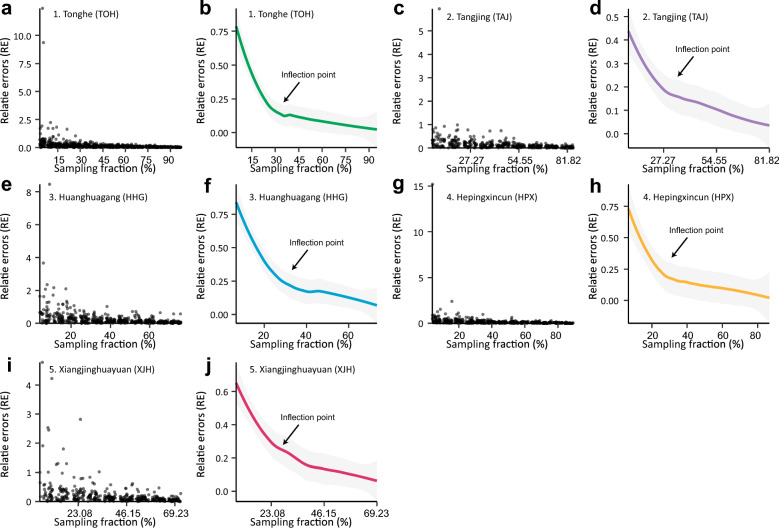
Relative errors (RE) vary with sampling fractions. (a, c, e, g, i) Raw data of ER wary with sampling fractions at five disctinct sites including Tonghe (**a**), Tangjing (**c**), Huanghuagang (**e**), Hepingxincun (**g**), and Xiangjinghuayuan (**i**). (b, d, f, h, j) Loess fitting of ER wary with sampling fractions at five disctinct sites including Tonghe (**b**), Tangjing (**d**), Huanghuagang (**f**), Hepingxincun (**h**), and Xiangjinghuayuan (**j**)

The data resampling tests across sampling fractions were conducted at five distinct sites, yielding 429, 462, 741, 351, and 351 sampling results, respectively. As the sampling fractions increased, all five sites demonstrated a gradual decrease in relative efficiency (RE) (Fig. 4). By applying loess fitting, we observed a clear inflection point ranging between 23% and 30% across all the sites (Fig. 4). Within the range of sampling fractions below the inflection point, the rate of RE significantly decreased (Fig. 4). Conversely, when the sampling fraction increased beyond this inflection point, RE began to increase at an accelerated rate (Fig. 4). Critically, increasing the inflection sampling fraction up to this point significantly enhanced the overall accuracy and stability of sample extrapolation. However, further increases beyond this inflection point had negligible effects on improving either overall accuracy or stability.

### Comparison of the monitoring effectiveness between IMT and MOT for *Ae. albopictus*

**Fig. 5:**
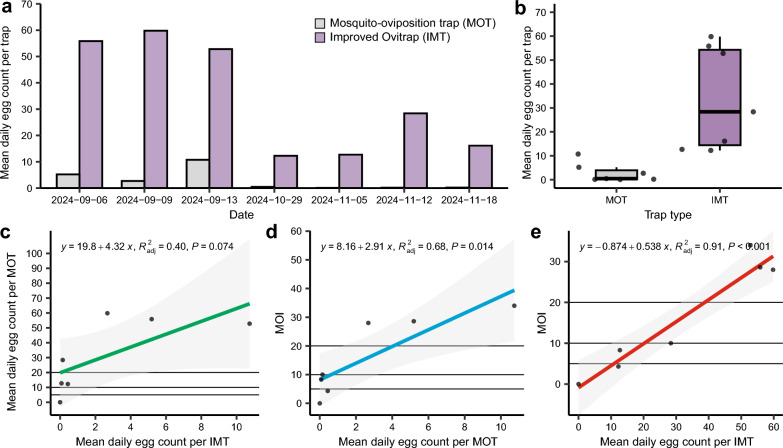
Comparison of the monitoring effectiveness of IMT and MOT for *Ae. albopictus*. **a** Temporal dynamics of egg number per trap for the IMT and MOT. **b** Comparison of the mean daily egg count per trap between the IMT and MOT. **c** Correlation analysis of the mean daily egg count between IMT (new ovitrap index, NOI) and the mean daily egg count per MOT. **d** Correlation analysis between the mosquito or oviposition positive index (MOI) and the mean daily egg count per MOT. **e** Correlation analysis between the NOI and MOI

From September 2024 to November 2024, a total of 2978 and 197 eggs were collected using IMT and MOT, respectively (Fig. 5a). Among these, 30 IMTs and 34 MOTs were positive, resulting in positivity rates of 100% and 22.6%, respectively (Fig. 5a). On average, each positive ovitrap captured 29.98 ± 22.89 eggs/day for the IMT and 2.43 ± 3.82 eggs/day for the MOT (Fig. 5a). Compared with the MOT, the IMT presented significantly higher positivity rates and consistently attracted more eggs (*t* = 2.085, *P* < 0.001; Fig. 5b), indicating greater field efficiency in terms of egg collection, even in winter (Fig. 5).

The monitoring trends of IMT and MOT showed consistent patterns. In addition, we observed a significant correlation between their daily egg count per trap (*r*^2^ = 0.68, *P* = 0.007; Fig. 5c). Although the MOT monitors both egg and adult mosquito counts, its derived MOI employs the container positivity rate as its primary metric. We found a positive correlation between the MOI and MOT average daily egg collection (*r*^2^ = 0.90, *P* < 0.001; Fig. 5d).

The commonly utilized mosquito density indicators derived from monitoring techniques include three primary types: (1) immature stage indices (larvae and pupae), (2) weekly eggs per ovitrap, and (3) weekly female mosquito counts per sticky gravid trap.

IMT exhibited superior efficiency for collecting eggs of *Ae. albopictus* in the field, particularly in winter. Therefore, we developed the new oviposition index (NOI), which is calculated as the mean daily egg collection per IMT trap. Our findings revealed a significant correlation between NOI and MOI across various investigation sites in Guangzhou (*r*^2^ = 0.45, *P* = 0.041; Fig. 5e).

### Establishment of an IMT-based surveillance strategy for local dengue epidemic control

On the basis of China’s experience with the control of dengue outbreak vector mosquitoes, we propose an improved IMT-based monitoring strategy for local dengue epidemic control. This surveillance system functions with three critical parameters: a density index, an adaptive scheme for the sampling site and monitoring radius selection, and a suitable sampling period.

**Fig. 6:**
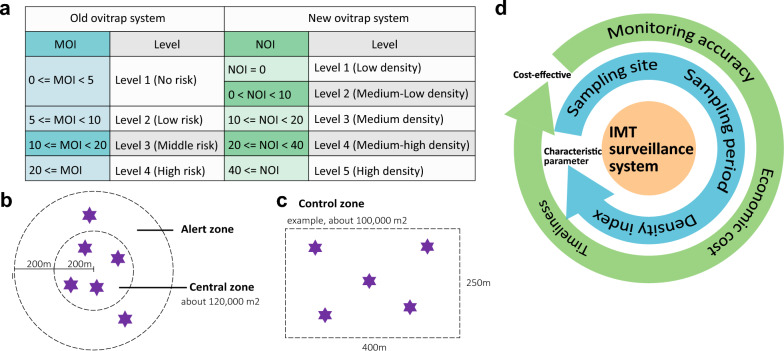
The classification scheme for monitoring mosquito density using the IMT and the scheme proposed for assessing vector mosquito density in epidemic areas. **a** Comparison of the classification schemes for IMT and MOT. **b**, **c** Proposed IMT setting in the central zone and alert zone during case-area targeted interventions (CATI). **d** The IMT-based surveillance strategy is utilized for efficacy evaluation for integrated vector management during outbreaks

Modern mosquito control operations increasingly depend on early warning systems equipped with evidence-based action thresholds to optimize intervention timing [33]. Two developed approaches for setting these thresholds include (1) meticulously planned systematic surveillance and investigation to understand associations between influential factors, mosquito populations, and local disease transmission dynamics; and (2) determining correlations between the new and existing indices. China currently uses MOIs with four control thresholds (< 5, 5–10, 10–20, and ≥ 20) for *Aedes*-borne disease control [24]. Our study revealed a significant linear relationship between the new NOI and the MOI (1 MOI unit is approximately equal to 2 NOI units). This finding enabled us to establish parallel NOI thresholds (0, 0–10, 10–20, 20–40, and ≥ 40) for vector control (Fig. 6a).

These thresholds enable timely planning and implementation of vector control measures. During an outbreak, a real-time rapid response scheme requires immediate intervention in core areas for optimal efficiency. The incorporation of expert recommendations for local disease control and rapid mosquito elimination (zero mosquito vector density) in outdoor core areas during early outbreaks is critical for epidemic control. Given NOI’s greater sensitivity, we designed the IMT-based surveillance strategy with two enhanced threshold levels (0 and 0–10; Fig. 6a), corresponding to an MOI level of 1 for stricter core area control.

China’s CATI program typically designates a 120,000 m^2^ core area for mosquito vector control. On the basis of our sampling fractions and monitoring radius estimates (Fig. 3), six IMTs sufficiently cover this core area. We recommend deploying four IMTs in the central case in the alert zone to increase monitoring efficacy (Fig. 6b). For areas differing from the standard size (100,000 m^2^), we propose (1) two to five IMTs for areas less than 100,000 m^2^ (adjusted for topography), and (2) one additional IMT per 100,000 m^2^ increment for larger areas (Fig. 6c). All deployments should prioritize vector breeding habitats for maximum efficiency (Fig. 6c).

The improved IMT strategy uses the NOI as the density index, typically deploying six IMTs per 120,000 m^2^. For vector management during outbreaks, we recommend three key operational points: first, the IMT should be deployed in 24-h cycles from 09:00 to 09:00 the following day. Second, traps should be placed at ground level in areas identified as potential mosquito breeding and resting sides. Third, optimal placement requires positioning the devices (particularly beneath tree canopies) while maintaining sufficient distance from areas of human/animal activity.

### Preliminary implementation of an IMT-based surveillance strategy within CATI practices

We conducted preliminary field testing of the IMT strategy during three CATI outbreak responses in Guangdong Province in 2024, with complete methodological details provided in Text S1. Throughout these four CATI operations, we standardized the mosquito egg collection frequency at daily intervals, sustaining continuous monitoring until local health authorities officially terminated each outbreak response. Our findings demonstrated that IMT-based surveillance remained operationally feasible across all three outbreak locations while effectively supporting the performance assessment of CATI-implemented integrated vector management strategies (Text S1).

## Discussion

Compared with advanced trapping systems, such as the BG-Sentinel trap [34], BG-Counter, MS-300 [35], and MK [36], which leverage advanced techniques such as the IoT, the IMT represents a cost-effective alternative that is particularly valuable. From an economic perspective, it is essential to assess the procurement and maintenance costs of these technologies. This is particularly crucial given that dengue is mainly a disease associated with poverty and has a relatively high prevalence in developing countries [37]. In contrast, economically advanced countries or cities are moving towards automated, digitized mosquito monitoring systems (Fig. 6d). Nevertheless, this advancement process necessitates continuous refinement and improvement over an extended period (Fig. 6d). Each monitoring system has a distinct advantage in addressing specific issues. For example, the IMT-based surveillance strategy provides essential support for evaluating the effectiveness of integrated vector management programs, effectively complementing existing MOT systems (Fig. 6d). However, our field experiments revealed limitations, particularly regarding incomplete alleviation of the position effect, indicating the need for continued in-depth research on the IMT across diverse locations and sampling points to optimize IMT implementation.

Within CATI frameworks for dengue outbreaks, we systematically developed an IMT-based surveillance strategy using the NOI to assess *Aedes* population density. Our results demonstrate that deploying six IMTs across a 120,000 m^2^ CATI area with 24-h period monitoring cycles effectively suppresses the outbreak response until termination (typically 25 case-free days). This approach has successfully supported three CATI initiatives controlling dengue transmission in Guangdong Province (2024), although urban spatial complexity—including vertical dispersion via high-rise elevators [33, 38] and year-round breeding in semi-enclosed spaces such as parking garages—necessitates further optimization of deployment density, frequency, and duration across various settings (ports, airports, and parks).

The transition of vector indices from theoretical predictors to practical early-warning tools has been significant. Although China’s existing MOI-based system classifies risk into four levels, the NOI offers superior responsiveness for CATI because of its daily monitoring capability, minimal trap requirements, and enhanced sensitivity. Our findings reveal a strong linear relationship between the NOI and MOI, enabling the development of parallel NOI thresholds (0, 0–10, 10–20, 20–40, and ≥ 40) for vector control grading. However, dengue transmission risk involves complex multifactorial dynamics—our prior MOI-based R_0_ model demonstrated monthly and temperature-dependent risk variations in Guangzhou [39]. The incorporation of NOI into a predictive model will require the integration of additional epidemiological factors through ongoing research.

## Conclusions

In this study, we developed and evaluated an IMT system for the surveillance of the population density of the dengue vector *Ae. albopictus* in Guangdong Province, China. Through systematic field testing, we optimized the design of the IMT and established its key operational parameters, including the effective working radius and optimal deployment density. Furthermore, we developed a comprehensive implementation strategy for integrating IMTs into CATIs, thereby introducing the NOI as a practical metric for assessing *Aedes* mosquito density. Our study revealed a linear relationship between NOI and MOI, suggesting similar grading standards for *Aedes* control using NOI. Preliminary field applications confirmed the IMT’s effectiveness in outbreak settings, providing crucial support for CATI operations. As a cost-effective and operationally efficient alternative to existing surveillance methods, the IMT system represents a significant advancement in dengue vector monitoring and is particularly suitable for resource-limited settings where sophisticated trapping systems may be impractical.

## Supplementary Information

Additional file 1. Fig. S1 Design and appearance of the improved Mosquito Ovitrap (IMT).

Additional file 2. Fig. S2 Temporal variations in the density map of *Ae. albopictus* populations monitored using IMTs.

Additional file 3. Fig. S3 Improved Mosquito Ovitraps (IMTs) monitoring radius determined using incremental spatial autocorrelation analysis. (a, b) Dynamics of Moran’s *z*-scores derived from incremental spatial autocorrelation analysis, based on *P*-value thresholds of 0.1 and 0.05, respectively. (c, d) Dynamics of the *P*-value obtained from incremental spatial autocorrelation analysis, using *P*-value thresholds of 0.1 and 0.05, respectively.

Additional file 4. Text S1 Report of the IMT-based surveillance application in the Shenzhen dengue epidemic CATI.

## Data Availability

Data supporting the conclusions of this article are included within the article and its additional files.
