# Supplementary figures and images for: An improved ovitrap-based surveillance framework: facilitating cost-efficient monitoring and efficacy assessment of integrated vector management strategies for dengue outbreak control
Source: Parasit Vectors. 2025 Sep 24;18:380. doi: 10.1186/s13071-025-07002-8 (PMC12462179; doi:10.1186/s13071-025-07002-8)

**a**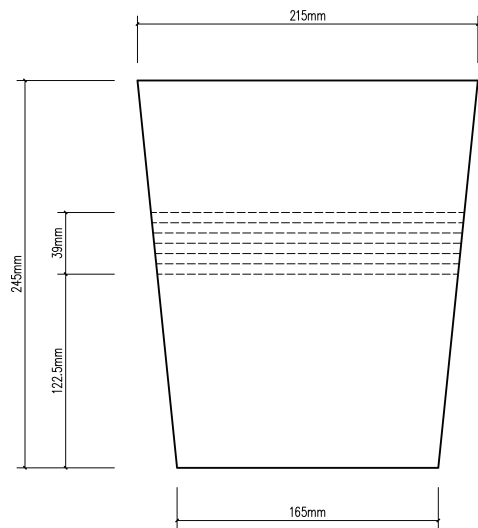**b**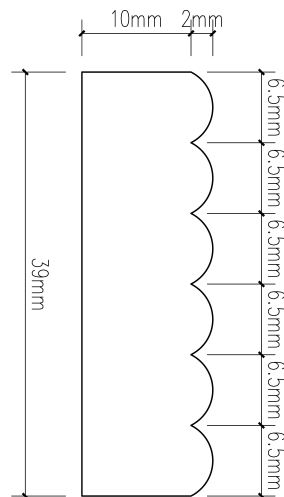**c**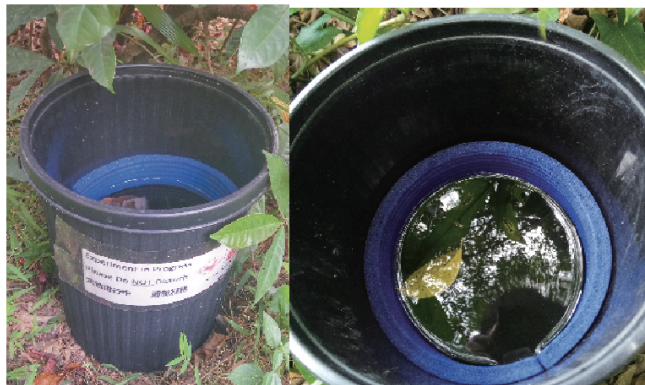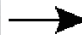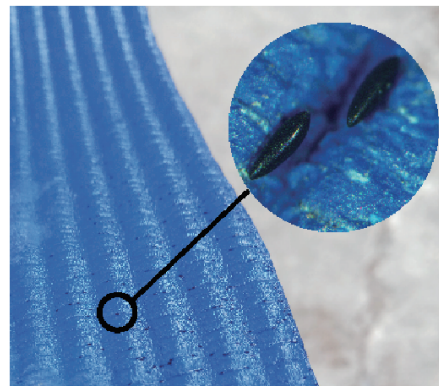

Supplement: Supplementary file 1 — Additional file 1. Fig. S1 Design and appearance of the improved Mosquito Ovitrap (IMT). [file 13071_2025_7002_MOESM1_ESM.pdf]

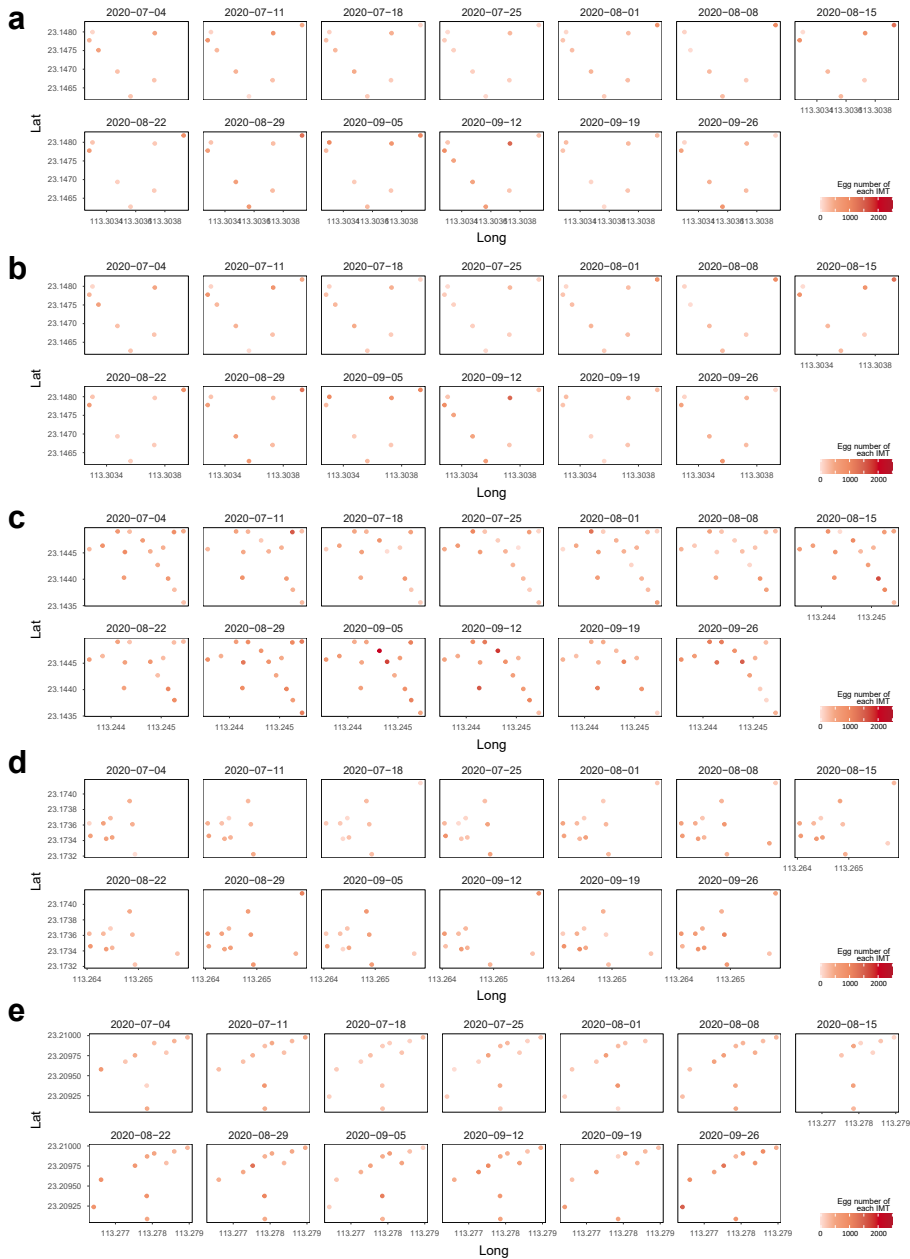

Supplement: Supplementary file 2 — Additional file 2. Fig. S2 Temporal variations in the density map of Ae. albopictus populations monitored using IMTs. [file 13071_2025_7002_MOESM2_ESM.pdf]

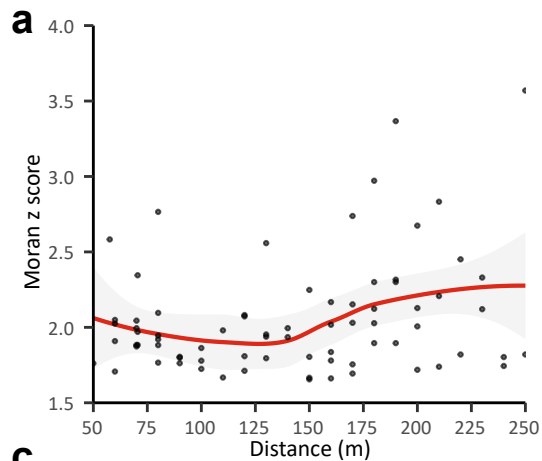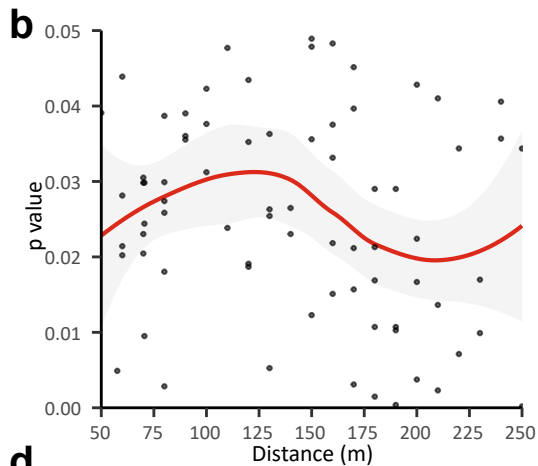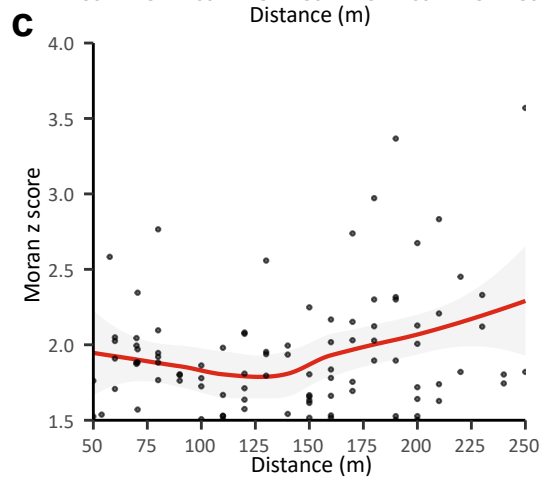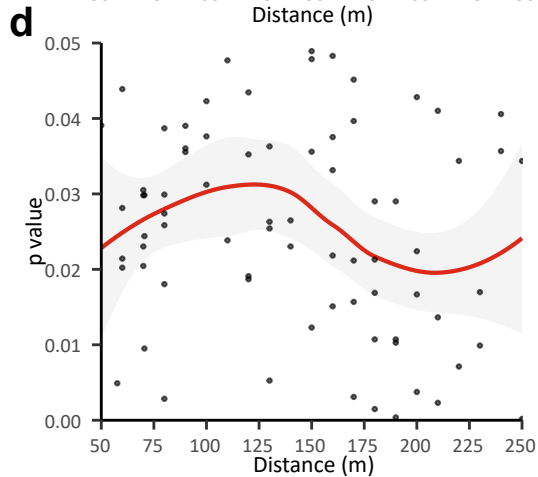

Supplement: Supplementary file 3 — Additional file 3. Fig. S3 Improved Mosquito Ovitraps (IMTs) monitoring radius determined using incremental spatial autocorrelation analysis. (a, b) Dynamics of Moran’s z-scores derived from incremental spatial autocorrelation analysis, based on P-value thresholds of 0.1 and 0.05, respectively. (c, d) Dynamics of the P-value obtained from incremental spatial autocorrelation analysis, using P-value thresholds of 0.1 and 0.05, respectively. [file 13071_2025_7002_MOESM3_ESM.pdf]
