# Supplementary material for: An improved ovitrap-based surveillance framework: facilitating cost-efficient monitoring and efficacy assessment of integrated vector management strategies for dengue outbreak control
Source: Parasit Vectors. 2025 Sep 24;18:380. doi: 10.1186/s13071-025-07002-8 (PMC12462179; doi:10.1186/s13071-025-07002-8)
Supplement: Supplementary file 4 — Additional file 4. Text S1 Report of the IMT-based surveillance application in the Shenzhen dengue epidemic CATI. [file 13071_2025_7002_MOESM4_ESM.pdf]

S1 Text. Report of IMT-based surveillance application in Shenzhen dengue epidemic CATI.

1. Introduction

From October to November 2024, four dengue fever outbreaks occurred in Luohu District of Shenzhen City. The affected sites were located at the construction site of Plot A4 in Hubei New Town (2024-10-21), the construction site of the Jingfu Garden Community (2024-10-05), and the construction site of Shawan Second Waterworks Phase 2 (2024-11-03).

The monitoring results indicated that following the implementation of disinfection measures, the mosquito density significantly decreased to a low level, corresponding to a NOI of Level 1 and a low risk classification. After 25 consecutive days of surveillance with no reported cases, monitoring activities were temporarily halted.

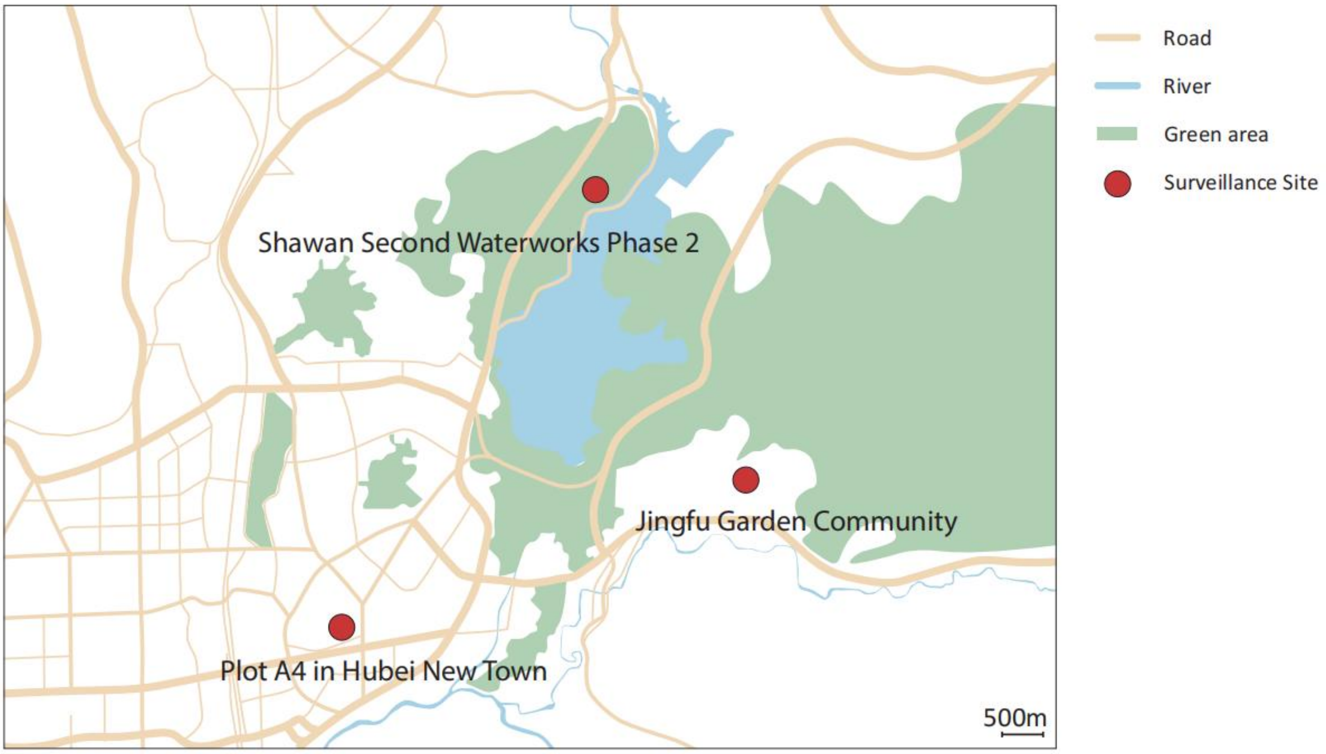

2. IMT surveillance in construction site of Plot A4 in Hubei New Town

2.1 Surveillance set

The site of A4 construction site in Hubei New Village is located on the northeast side of the intersection of Hubei Road and Leyuan Road, Dongmen Street, Luohu District, surrounded by complex residential living areas. The monitoring area is the construction area, the construction materials are accumulated, and the waste materials are accumulated. The construction site area is approximately about 80,000 m^2, and 5 IMTs were set.

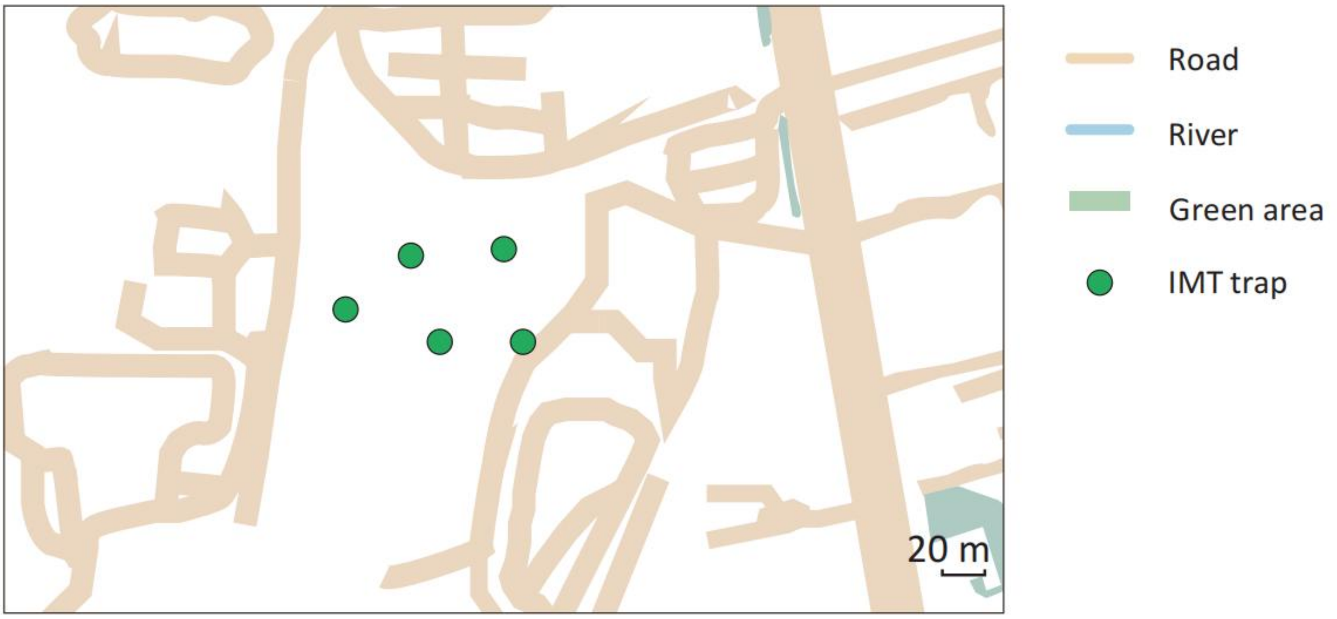

2.2 Surveillance results

Date	Status	Egg number	NOI	Level
2024-10-24	Normal ☑ Malfunctioned ☐	0	1	1
2024-10-25	Normal ☑ Malfunctioned ☐	0	1	1
2024-10-26	Normal ☑ Malfunctioned ☐	0	1	1
2024-10-27	Normal ☑ Malfunctioned ☐	0	1	1
2024-10-29	Normal ☑ Malfunctioned ☐	0	1	1
2024-10-30	Normal ☑ Malfunctioned ☐	0	1	1
2024-10-31	Normal ☑ Malfunctioned ☐	0	1	1
2024-11-01	Normal ☑ Malfunctioned ☐	0	1	1
2024-11-03	Normal ☑ Malfunctioned ☐	0	1	1
2024-11-04	Normal ☑ Malfunctioned ☐	0	1	1

3. IMT surveillance in Jingfu Garden Community

3.1 Surveillance set

Since August 8, 2022, Jingfu Garden Project has obtainedobtained the Shenzhen Construction Project Planning Permit with an area of about 17133.56 square meters. The site of Jingfu Garden Project in Hubei New Village is located on the southwest side of the intersection of Guowei Road and Panshan Road in Liantang Street, Luohu District, surrounded by complex residential living areas. The construction site area is approximately about 17,000 m^2, and 5 IMTs are set.

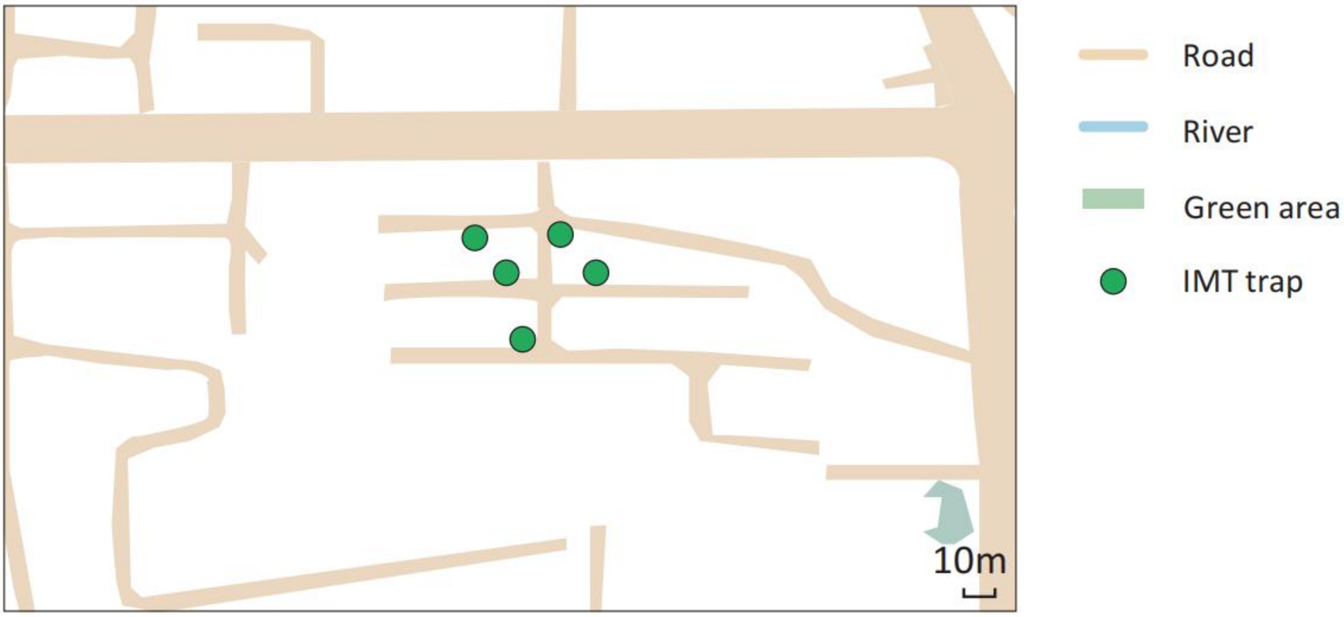

3.2 Surveillance results

Date	Status	Egg number	NOI	Level
2024-11-06	Normal ☑ Malfunctioned ☐	0	0	1
2024-11-07	Normal ☑ Malfunctioned ☐	0	0	1
2024-11-08	Normal ☑ Malfunctioned ☐	0	0	1
2024-11-10	Normal ☑ Malfunctioned ☐	0	0	1
2024-11-11	Normal ☑ Malfunctioned ☐	0	0	1
2024-11-12	Normal ☑ Malfunctioned ☐	0	0	1

4. IMT surveillance in construction site in Shawan Second Waterworks Phase 2

4.1 Surveillance set

The construction site area is about 80,000 m^2, and 5 IMTs were set.

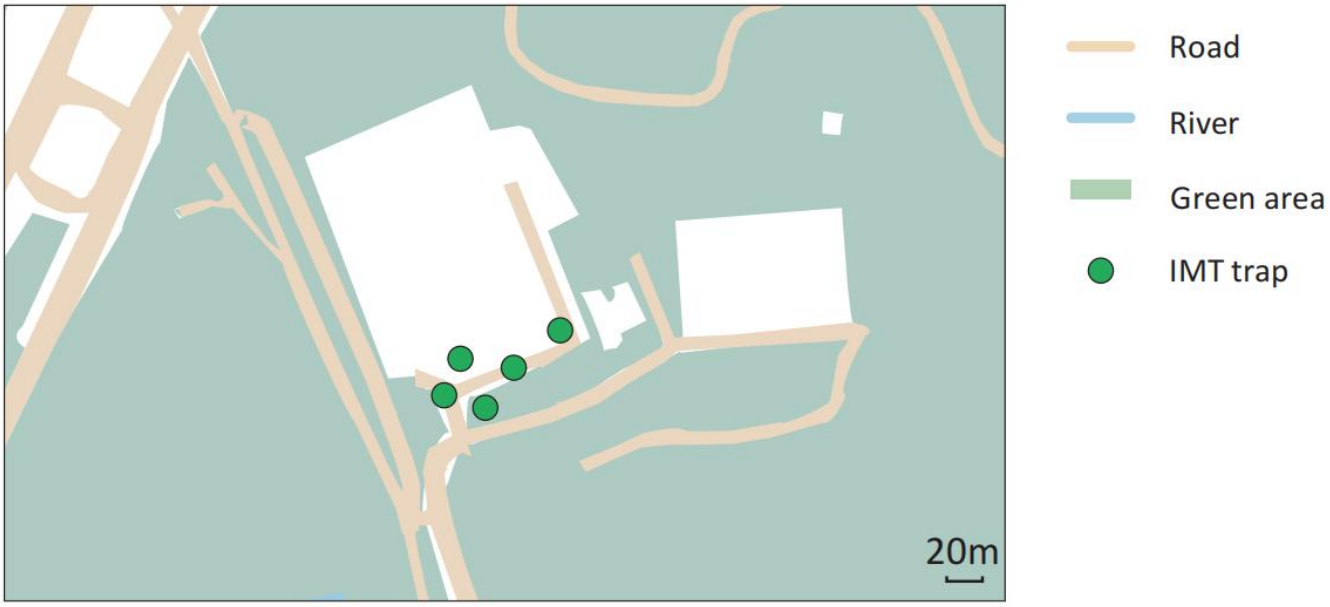

4.2 Surveillance results

Date	Status	Egg number	NOI	Level
2024-11-25	Normal ☑ Malfunctioned ☐	11	2.2	2
2024-11-26	Normal ☑ Malfunctioned ☐	0	0	1
2024-11-27	Normal ☑ Malfunctioned ☐	0	0	1
2024-11-28	Normal ☑ Malfunctioned ☐	0	0	1
2024-11-29	Normal ☑ Malfunctioned ☐	0	0	1
2024-12-02	Normal ☑ Malfunctioned ☐	2	0.4	2
2024-12-03	Normal ☑ Malfunctioned ☐	13	2.6	2
2024-12-04	Normal ☑ Malfunctioned ☐	0	0	1
